# Enhanced Lipid Productivity and Photosynthesis Efficiency in a *Desmodesmus* sp. Mutant Induced by Heavy Carbon Ions

**DOI:** 10.1371/journal.pone.0060700

**Published:** 2013-04-09

**Authors:** Guangrong Hu, Yong Fan, Lei Zhang, Cheng Yuan, Jufang Wang, Wenjian Li, Qiang Hu, Fuli Li

**Affiliations:** 1 Shandong Provincial Key Laboratory of Energy Genetics, Qingdao Institute of Bioenergy and Bioprocess Technology, Chinese Academy of Sciences, Qingdao, PR China; 2 Institute of Modern Physics, Chinese Academy of Sciences, Lanzhou, PR China; 3 Laboratory for Algae Research and Biotechnology (LARB), College of Technology and Innovation, Arizona State University, Mesa, Arizona, United States of America; Belgian Nuclear Research Centre SCK/CEN, Belgium

## Abstract

The unicellular green microalga *Desmodesmus s*p. S1 can produce more than 50% total lipid of cell dry weight under high light and nitrogen-limitation conditions. After irradiation by heavy ^12^C^6+^ ion beam of 10, 30, 60, 90 or 120 Gy, followed by screening of resulting mutants on 24-well microplates, more than 500 mutants were obtained. One of those, named D90G-19, exhibited lipid productivity of 0.298 g L^−1^⋅d^−1^, 20.6% higher than wild type, likely owing to an improved maximum quantum efficiency (Fv/Fm) of photosynthesis under stress. This work demonstrated that heavy-ion irradiation combined with high-throughput screening is an effective means for trait improvement. The resulting mutant D90G-19 may be used for enhanced lipid production.

## Introduction

Under suitable environmental conditions, microalgae synthesize fatty acids mainly for the production of membrane glycerolipids, such as glycolipids and phospholipids. However, under unfavorable growth conditions, many microalgae change their lipid biosynthetic pathways to produce large amounts of neutral lipid (20–50% of cell dry weight), mostly in the form of triacylglycerol (TAG), which mainly are stored in cytosolic lipid bodies [1]. Recently, oleaginous microalgae have been regarded as potential next-generation feedstocks for biofuels (e.g., biodiesel and jet fuel) because they exhibit higher photosynthetic efficiency and a greater lipid production rate than terrestrial oil crops [2]–[4]. Microalgae do not compete for precious arable land with grain crops and many species can be cultured in wastewater or salt water [2]. However, naturally occurring microalgae that have been used so far produce much lower amounts of neutral lipid than the theoretical maximum [1]. Many methods such as physical or chemical mutagenesis and genetic engineering tools may be applied to the production strains for improving lipid production [5]–[7].

In recent years, some genetic engineering approaches have been applied to several algal species aiming at improving lipid production, including *Chlamydomonnas reinhardtii* [8], [9], *Phaeodactylum tricornutum* [10], *Cyclotella cryptica* [11], and *Nannochloropsis* sp. [5]. However, only moderate success was achieved, mainly because the lipid metabolism, particularly the functions of key genes and enzymes involved in lipid synthesis and accumulation in these organisms, is not well understood. Furthermore, some transcription factors involved in the regulation of plant lipid accumulation were suggested as the second-generation targets to improve the lipid contents [12]–[14].

On the other hand, various physical mutagens have been successfully applied to crop breeding and genetic manipulation of microorganisms for trait improvement. As a physical irradiation source, heavy-ion beam induces a broad range of mutations i.e. base substitutions, small and large insertions/deletions, translocation and inversions in the genomes of microalgae. The use of a heavy-ion beam as mutagen has the following advantages: (1) It has a high linear energy transfer (LET) with energetic heavy ions that can produce dense ionization along their trajectories, and cause complex and irreparable damages of DNA. Hence mutations induced by heavy-ion may show a broad spectrum and a high frequency; (2) it causes higher relative biological effectiveness (RBE) compared with low-LET γ-rays and x-rays [15]; (3) it can be controlled so as to deposit high energy at precise positions, in contrast to low-LET irradiation that may cause large deletions, translocations or rearrangements in the genome of a given organism [16]. Like other random mutagenesis methods, heavy-ion irradiation can generate thousands of mutants, but it is a very laborious work to screen for desirable mutants. Establishment of a high-throughput screening method will be helpful for microalgal trait improvements.

The green microalga *Desmodesmus* sp. (Sphaeropleales, Scenedesmaceae) S1 strain was oval, 5–10 µm in length. It had a thin, rim-like, pyrenoid-containing chloroplast that enlarged with age. It was once identified as *Pseudochlorococcum* sp. [17]. However, it should be a strain in the genus *Desmodesmus* according to its phylogenetic relationship with 22 Chlorophyta taxa based on their ITS sequences [18]. *Desmodesmus* sp. S1 exhibited rapid growth and reproduction while possessing high lipid content (up to 55% of dry weight) in BG-11 culture medium. The preliminary experiments showed that a high biomass productivity of 0.5 g L^−1^⋅d^−1^ can be achieved in *Desmodesmus* sp. S1 cultures under stress conditions, which was superior to many microalgal strains used for biofuel production [1], [2].

In this paper, we introduced a heavy-ion irradiation method to induce mutagenesis of *Desmodesmus* sp. A large number of *Desmodesmus* mutants were screened by coupling a chlorophyll fluorometer with a Nile red staining method. We demonstrated that a mutant D90G-19 exhibited a higher photosynthetic efficiency and higher lipid content, thus resulting in higher lipid productivities than WT.

## Materials and Methods

### Strains and Culture Conditions

*Desmodesmus* sp. S1 strain was isolated in Arizona, USA and provided by Professor Qiang Hu at Arizona State University, ASU. The wild type (WT) and mutant seed cultures were grown in BG-11 culture medium in a column photobioreactor (60 cm high, diameter 4 cm, 300 mL culture volume) under continuous illumination of a low light intensity of 50 µmol photons m^−2^⋅s^−1^. Culture mixing was provided by aeration with compressed air containing 2% CO_2_. This treatment was referred to as LL+N. After 4 days, the seed culture were collected by centrifugation (3200 ×g, 5 min) and transferred into 300 mL or 10 L nitrogen-limited BG-11 medium (4.25 mM NaNO_3_) in column or panel photobioreactors, respectively. The initial OD_750_ of algal culture was about 0.2. WT and mutant cultures were grown under continuous illumination of 400 µmol photons m^−2^⋅s^−1^ with aeration containing 2% CO_2_ (which was referred to as HL-N) for two weeks. All strains were cultured in 3 replicates at room temperature (25±2°C).

Pre-weighed Whatman GF filter paper (Whatman International Ltd., Maidstone, UK) was recorded as W1. A 10 mL culture sample was filtered through the pre-weighed filter paper which was then dried at 105°C for at least 8 hours. Final weight of the filter paper was recorded as W2. The difference between W1 and W2 was the dry weight of algal biomass.

Microalgal suspension was passed through a filter paper (0.22 µm) to remove cells, and nitrate in the filtrate was analyzed according to the method of Collos [19].

### Heavy-ion Irradiation

*Desmodesmus* sp. S1 strain was maintained in BG-11 medium in 100 mL Erlenmeyer flask under low light illumination of 100 µmol photons m^−2^⋅s^−1^ at room temperature for 3 days. Algal cells at the exponential growth phase were collected by centrifugation (3200 ×g, 3 min) and washed with sterile water, then resuspended in fresh BG-11 medium. The cell concentration was adjusted to 1×10^6^ cells⋅mL^−1^, and exposed to ^12^C^6+^ ion beam provided by the Heavy Ion Research Facility at Lanzhou (HIRFL), Institute of Modern Physics of Chinese Academy of Sciences (CAS). Irradiation treatments were conducted at dosages of 10, 30, 60, 90 and 120 Gy, calculated from particle fluencies and LET, and there were at least three algae samples for every dose treatment.

### Mutant Isolation

After irradiation, algae cells were plated on BG-11 agar plates in triplicate and cultured at 25°C under low light (50 µmol photons m^−2^⋅s^−1^) until algal colonies occurred on the plates. The colonies derived from the irradiated cells were selected and transferred to BG-11 agar plates several times to obtain purified monoclonal strains, which were regarded as putative mutants and constituted the mutant library.

### Analysis of Photosynthesis Efficiency and Oxygen Evolution Rate

Algal samples (cells suspension or colonies on plate) were placed in a chamber of the Imaging-PAM (pulse-amplitude) Chlorophyll Fluorometer (Walz, Effeltrich, Germany), and the Fv/Fm values of samples were determined. Three replicates for each mutant were analyzed.

Two milliliter of algal suspension was placed in the chamber of Chlorolab 2 (Hansatech Instruments Ltd., King’s Lynn, UK), and the oxygen evolution rate was determined according to the manufacturer’s instructions.

### Screening of Lipid and Photosynthesis Mutants

The putative mutants and wild type were inoculated into 24-well microplates where each well contained 2.5 mL BG-11 culture medium, and cultured under low light (<100 µmol photons m^−2^⋅s^−1^) at 25°C for 6 days. At the sixth day, OD_750_ of the cultures were measured with a spectrophotometer (UV-2600, Unico Instruments Co., Ltd., Shanghai, China). The 24-well microplates were subjected to a high light intensity of 300 µmol photons m^−2^⋅s^−1^ for 6 more days. The neutral lipid content of mutants and wild type was determined by a modified protocol of Chen [20]. Ten microliter Nile red solution (125 µg⋅mL^−1^ in acetone) was added into individual wells containing 2.0 mL of cultures. The 24-well microplates were vortexed for 1 min and incubated at 40°C for 10 min. After the algal cells were stained, fluorescence emissions were recorded with Synergy HT (Biotek Instruments Inc., Winooski, VT) with the excitation and emission wavelengths of 490 nm and 580 nm, respectively. Three replicates for each mutant were analyzed. As the Nile red staining method was microalgae species and physiological state sensitive [20], the linear regression equation (y = (x−21.98)×4.45) and its correlation coefficient of R^2^ (0.972) between Nile red fluorescence intensity (x) and lipid concentration (y: µg⋅mL^−1^) of *Desmodesmus* sp. S1 had been determined before using the Nile red fluorescence method. Then the data of lipid concentration were converted into a percentage of dry weight of algal biomass.

The putative mutants were also inoculated into BG-11 plates and cultured under light (200 µmol photons m^−2^⋅s^−1^) at 25°C. After 6 days, the Fv/Fm values of algal mutants were analyzed with an Imaging-PAM Chlorophyll Fluorometer (Walz, Effeltrich, Germany). Three replicates for each mutant were analyzed.

### Lipid Extraction, Quantification and Separation

Total lipids were extracted and quantified according to the Bigogno’s method [21] with minor modifications.

Total lipids were further separated by column chromatograph using a silica gel (60–200 mesh, Sigma-Aldrich, St. Louis, MO, USA) according to the following procedures: 100 mL chloroform, acetone and methanol were used as the eluent to collect the neutral lipid class, glycolipids and phospholipids, respectively. After evaporating the solvents using a rotary evaporator (RE-52AA, Yarong Inc., Shanghai, China), the remaining individual lipid fractions were dissolved in chloroform and transferred into pre-weight vials. Chloroform was removed using nitrogen evaporator and the residuals were freeze-dried for at least 12 h and weighed. The difference between the final weight and the weight before freeze-drying was the weight of lipid fractions.

Total lipid, neutral lipid, glycolipid or phospholipid were transmethylated with 2% H_2_SO_4_ in methanol at 85°C for 2.5 h. Heptadecanoic acid (C17∶0, 3 mg⋅mL^−1^) was used as an internal standard. Gas chromatograph analysis was performed with a GC system (7890A, Agilent technologies, Inc. CA).

### Pigments and Starch Analysis

The algal pellets were ground with quartz sands in liquid nitrogen until algal cells were broken completely. Then the pellets were ground with 2–3 mL ice-cold acetone and repeated for 2–3 times. All solutions were collected. After centrifugation at 3200×g for 5 minutes, the supernatant was transferred to a new tube. The acetone was evaporated under nitrogen gas and the residuals were lyophilized for 5 hours. Afterwards, 5 mL 80% acetone was added, and the solution absorbance at 663 nm, 646 nm and 470 nm were measured with a spectrophotometer (UV-2600, Unico Instruments Co., Ltd., Shanghai, China, resolution range: 4 nm). The contents of chlorophyll a, chlorophyll b and carotenoid were calculated based on the equation of Wellburn [22].

The starch content was analyzed with the starch assay kit (SA20, Sigma-Aldrich, St. Louis, MO). About 0.1–0.5 gram microalgal sample was ground into powder in liquid nitrogen, then transferred into a flask. After adding 25 mL deionized water, the solution was adjusted to pH of 5–7, and boiled for 3 minutes with gentle stirring. Afterwards, the solution was autoclaved at 135°C for 1 hour. When the temperature of the solution was reduced to about 60°C, deionized water was added to make a total volume of 100 mL. The extracted starch was hydrolyzed to glucose by amyloglucosidase. After subsequent phosphorylation by hexokinase and ATP, the glucose-6-phosphate was oxidised to 6-phosphogluconate by glucose-6-phosphate dehydrogenase in the presence of nicotinamide adenine (NAD). During this reaction, an equimolar amount of NAD was reduced to NADH, which resulted in the consequent increase in absorbance at 340 nm that was directly proportional to the glucose concentration. The starch concentration (µg⋅mL^−1^) was determined according to the formula described in the kit, and was converted into a percentage of dry weight of biomass.

### Statistical Analysis

A two-tailed paired t-test was applied to ascertain significant differences using SPSS (Statistical Product and Service Solutions) version 10.0 (SPSS Inc., Shanghai, China) and the level of statistical significance was P<0.05.

## Results and Discussion

### Lethality, Mutation Frequency of the Microalgal Cells Irradiated by ^12^C^6+^ Ion Beam, and Mutants Screening

**Figure 1:**
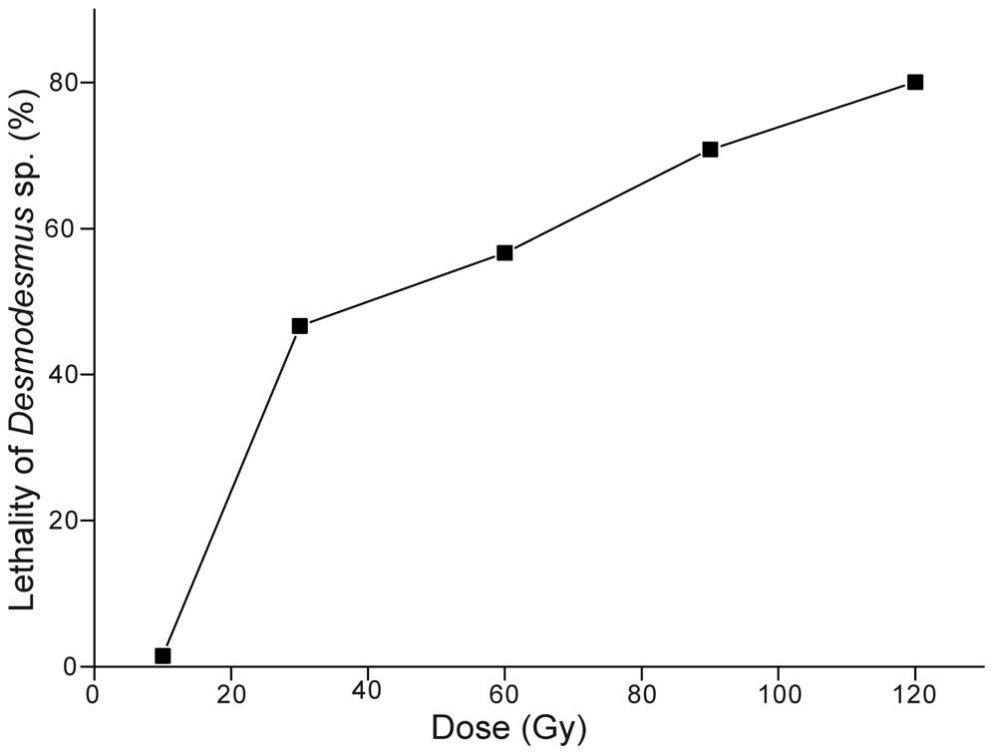
Effects of irradiation on the cells viability of *Desmodesmus* sp. S1.

The lethality of *Desmodesmus* sp. S1 after high-LET heavy ^12^C^6+^ ion beam irradiations was shown in Fig. 1. The relationship between the lethality of *Desmodesmus* sp. S1 and the irradiation dose of ^12^C^6+^ beam was fitted to a logistic curve equation (y = −22.20+21.09×ln(x−6.91), R^2^ = 0.98), which indicated the death rate of cells increased with increasing the radiation dosage from 10 to 120 Gy, with the highest lethality occurring between 90 to 120 Gy. The LD50 value was 37.57 Gy and the lethality of algal cells reached 80% at 120 Gy.

After heavy-ion beam treatment, the algae colonies that appeared on agar plates were considered putative mutants. A preliminary screening of the putative mutants by light microscopy showed that the morphological characteristics (e.g. colony appearance, cell shape and size etc.) of the putative mutants were indistinguishable from the wild type cells. Then photosynthetic characteristics (e.g., Fv/Fm, quantum efficiency of PSII) were used as alternative parameters to further characterize the putative mutants by using a chlorophyll fluorescence technique [23]. Previously, the chlorophyll fluorescence has been used as a sensitive, quantitative parameter to analyze the photosynthetic characteristics of cultivars [24]–[26] as well as to characterize microalgal mutants [27], [28].

Here, we applied a chlorophyll fluorometer Imaging-PAM to our mutant screening effort. The Imaging-PAM can measure several photosynthetic parameters (e.g., Fv/Fm) of 96 algal samples in a 96-well microplate within 10 minutes. Under favorable conditions (e.g., nutrient replete and low light), the Fv/Fm value of *Desmodesmus* sp. WT cells was 0.6–0.7. The colonies with significantly different Fv/Fm values from WT were identified as possible photosynthesis efficiency mutants (PEMs). The frequency of PEMs induced by 30, 60, and 90 Gy of ^12^C^6+^ beam were 14.5%, 25.8% and 28.5%, respectively. The dose of 90 Gy represented a trade off between cell survival rate and mutation frequency. As a result, about 500 mutants were obtained by this treatment.

Previous studies with terrestrial plant materials such as seeds, leaves and other organs indicated that the mutation rate ranged from 8.4% to 17.8% [15], [16], [29] when irradiated with heavy-ion beam, which was higher than that induced by traditional mutagenesis, such as x-ray, γ-ray or EMS [15]. The mutation rate of up to 28.5% in *Desmodesmus* sp.S1 induced by heavy-ion beam was considerably higher than those of plant materials, which implied that heavy-ion beam can be an effective method for mutagenesis of microalgae.

All colonies were also subjected to screening for lipid-overproduction mutants (LOMs) using a modified Nile red method in conjunction with the determination of growth rate, as indicated by optical density of algal culture measured at 750 nm.

**Figure 2:**
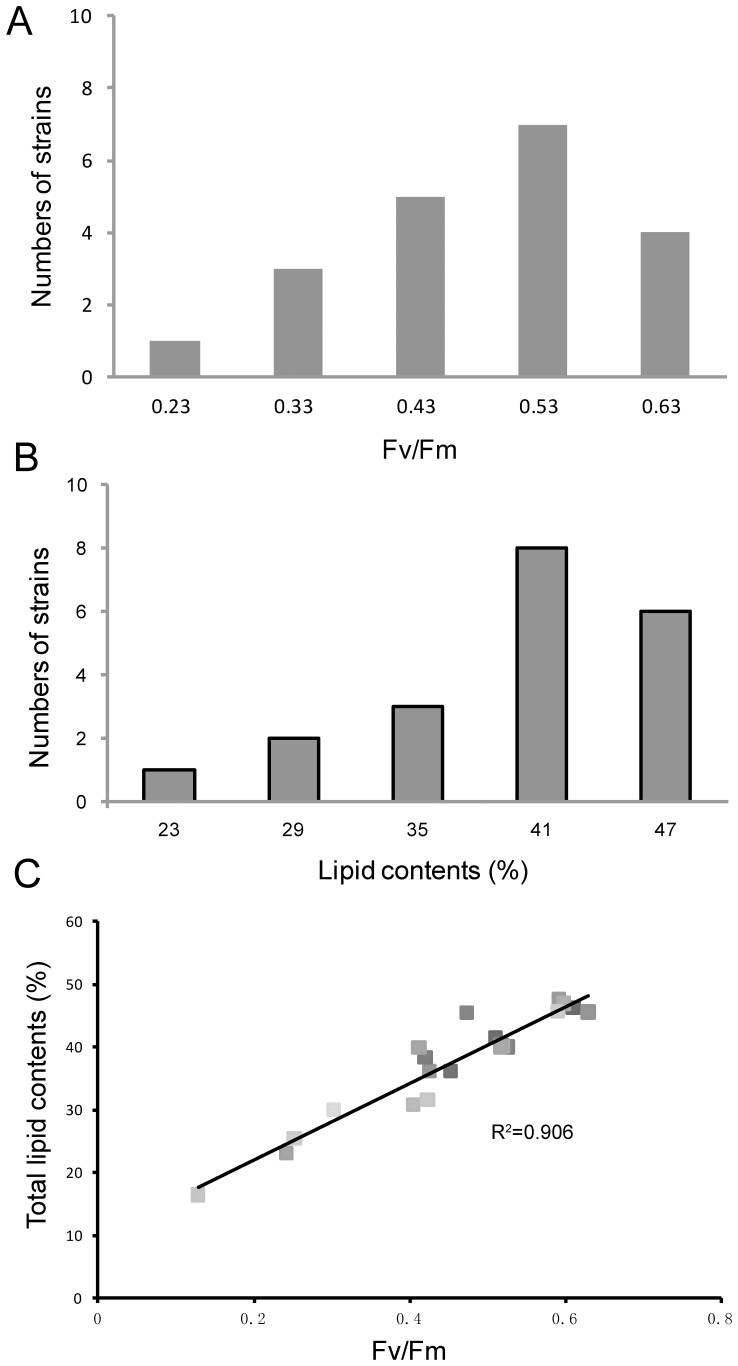
Distribution histogram of *Desmodesmus* sp. mutant phenotypes. (A) Distribution histogram of photosynthesis efficiency mutants (PEMs), (B) distribution histogram of lipid-over-production mutants (LOMs), and (C) the relationship between lipid contents and high light-adapted photosystem II efficiency under stress conditions in the PEMs and LOMs of *Desmodesmus* sp. S1. The distribution histograms of LOMs and PEMs were obtained using SPSS 10.0.

The mutants obtained through the first round screening with Imaging-PAM and Nile red fluorescence method would be subjected to the next rounds of screening. Among these mutants, a wide range of phenotypic distribution of PEMs was observed and approximately in line with the normal distribution (Fig. 2A). A similar normal distribution of phenotypes also occurred among the LOMs (Fig. 2B). In the following screening procedures, the lipid contents and photosynthetic efficiency of mutants were assayed when they were cultured in a column bioreactor. Lipid contents of many PEMs was significantly different with WT under stress conditions, thus were also identified as LOMs (Table S1), which indicated that heavy-ion irradiation induced mutations of unicellular microalgae had a wide spectrum and a high frequency as occurred to plants and mammalian cells [16], [30], [31]. A positive correlation (R^2^ = 0.906) existed between the lipid content and high light-adapted PSII efficiency (Fv/Fm) under stress conditions (Fig. 2C). Similar phenomena were also observed in naturally-occurring microalgae strains [32] and crop cultivars [33] because all carbohydrates including lipids in the photoautotroph were ultimately derived from photosynthesis. On the contrary, not every mutant with high Fv/Fm can produce high yields because the yields were also affected by other metabolic process such as respiration. We noted some mutants with higher Fv/Fm value than wild type that did not produce more biomass or lipids (data unpublished).

Current procedures for the screening of microalgae mutants that have a high yield of biomass and high lipid productivity suffer from two obstacles. Firstly, in unicellular microalgae morphological differences between wild type and most mutants are difficult to discern. Secondly, the quantification of the biomass and lipid contents of microalgae cultivated in conventional culture systems (e.g., flasks, carboys, bags, glass columns) is laborious, although some rapid detection methods such as the Nile red fluorescence method have become available. Therefore, we developed a new strategy to accelerate the screening of microalgae mutants with high yields of biomass and lipids based on the determination of the Fv/Fm value. All mutants were firstly subjected to the analysis of Fv/Fm value under different light intensities by Imaging-PAM. Afterwards, the biomass and lipid yields of those mutants with high Fv/Fm value were quantified in 24-well microplates. Only those mutants that have higher photosynthetic efficiency and lipid yields at least 10% greater than wild type were selected for further study, whereas the other mutants were discarded.

It would otherwise take at least 12 days to get these parameters analyzed for a mutant if it were cultured in a traditional culture system. Assuming that a maximum of 20 mutants could be tested every time, it will take at least 28 days to completely analyze 48 mutants. However, by applying the new strategy, analysis of 48 mutants can be achieved on six 24-well microplates within 8 days. Therefore, the efficiency of the new screening method was higher than that of traditional methods.

### Biomass, Total Lipid and Starch Contents of Wild Type and Mutant D90G-19 under HL-N

**Figure 3:**
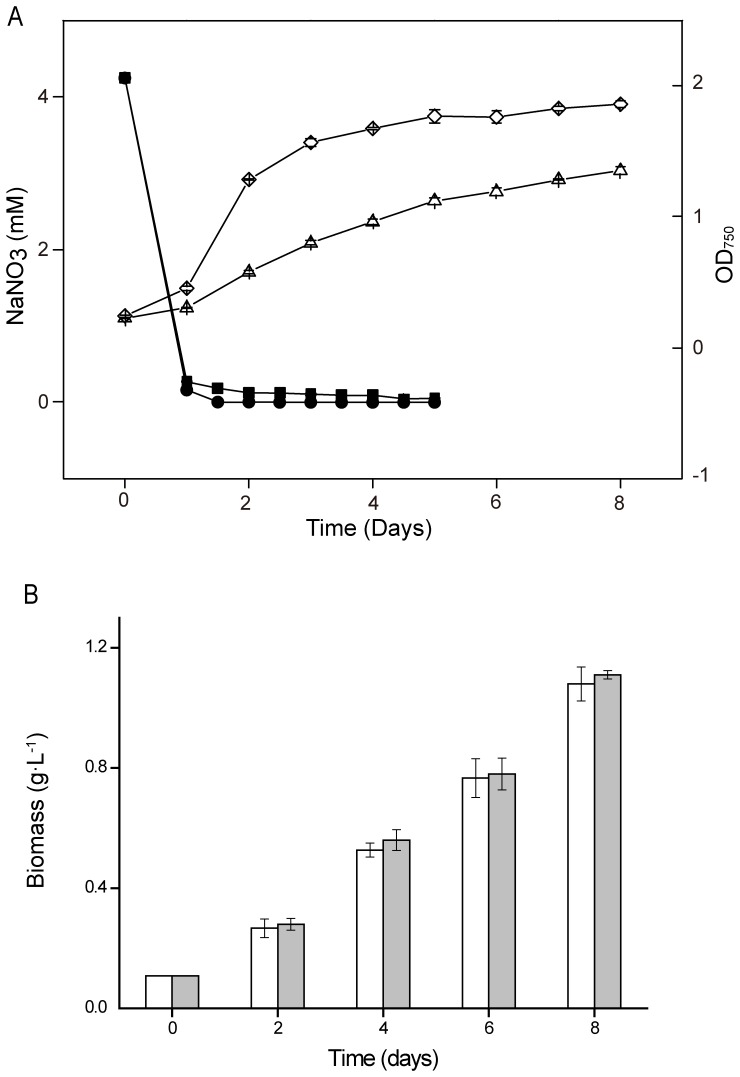
The consumption of NaNO_3_ and growth kinetics of *Desmodesmus* sp. (A) The consumption of NaNO_3_ in the nitrogen-limited (4.25 mM) BG-11 medium (closed symbols) and growth kinetics (open symbols) of *Desmodesmus* sp. S1 under low light (closed square, open triangle) and high light (closed circle, open diamond), and (B) biomass concentration of *Desmodesmus* sp. S1 wild type (WT, open square) and mutant D90G-19 (closed square) cultivated in BG-11 medium under LL+N conditions (50 µmol photons m^−2^⋅s^−1^, BG-11 medium with 17 mM NaNO_3_).

After 3–4 rounds of the screening process mentioned above, a mutant named D90G-19 with higher photosynthetic efficiency and lipid contents than WT was obtained. In a batch culture mode, once inoculated in a BG-11 medium (4.25 mM Nitrate) and exposed to light illumination, *Desmodesmus* sp. S1 grew rapidly for 4–5 days after a temporary lag phase (0–24 hours), and reached a stationary phase thereafter (Fig. 3A). During the first 2 days, almost all nitrates in the medium were consumed by algal cells. When WT and D90G-19 were cultivated under high light and nitrogen limitation (HL-N) conditions, the specific growth rates of WT and mutant D90G-19 were 0.598 and 0.630 g⋅L^−1^⋅d^−1^ in 8 days, respectively, and the maximum biomass (dry weight) concentration of 4.95 and 5.23 g⋅L^−1^ were obtained in the WT and D90G-19 cultures (Fig. S1), but the differences were not statistically significant. Under low light conditions, the mutant D90G-19 also showed the same biomass profile as wild type (WT) (Fig. 3B).

**Figure 4:**
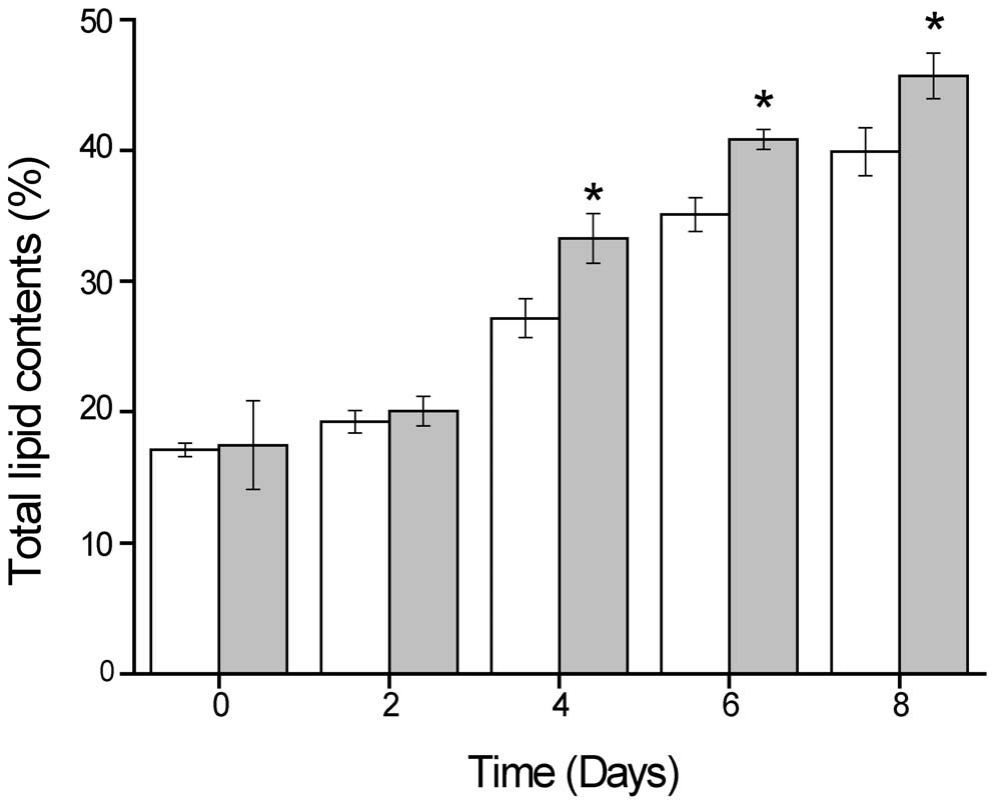
Total lipid contents of *Desmodesmus* sp. WT and D90G-19 were grown under HL-N conditions (300–400 µmol photons m^−2^⋅s^−1^, nitrogen-limited BG-11 medium with 4.25 mM NaNO_3_). The total lipid of the mutant D90G-19 was significantly higher than WT at day 4 (P = 0.0051), day 6 (P = 0.006) and day 8 (P = 0.0039). D90G-19: closed square; WT: open square.

While little difference in the lipid content was observed in WT and mutant D90G-19 grown under low light (LL+N), significantly higher amounts of total lipid were obtained in D90G-19 than in the WT when exposed to high light and under nitrogen limitation (HL-N) conditions (Fig. 4). As lipid production is a function of the lipid content and biomass concentration, the total lipid productivity of mutant D90G-19 was 0.298 g⋅L^−1^⋅d^−1^ during 8 days, which was 20.6% greater than that produced by WT (0.247 g⋅L^−1^⋅d^−1^).

In order to determine the stability of the phenotypic traits of D90G-19, it was cultivated in a 15 L panel photobioreactor under HL-N. It was shown that D90G-19 accumulated more lipids than WT (Fig. S2B), although the biomass of D90G-19 was comparable with that of WT (Fig. S2A). During 14 days, the total lipid productivity of D90G-19 was 0.105 g⋅L^−1^⋅d^−1^, and 17.1% greater than that of the WT (0.089 g⋅L^−1^⋅d^−1^), showing that the mutant D90G-19 had the potential for large scale biofuel production.

Total lipid includes neutral lipids, glycolipids and phospholipids along with small amounts of sterol esters and pigments. Neutral lipids are non-membrane lipids mainly in the form of triacylglycerols (TAGs) stored in oil droplets in the cytosol of the cells [34]. In *Desmodesmus* sp. WT, the concentrations of neutral lipid, glycolipid, and phospholipid on a per total lipid basis were 78.1%, 14.9% and 6.9%, respectively, when cultivated under HL-N for 8 days. Under the same conditions, the neutral lipid, glycolipid and phospholipid contents in D90G-19 were 87.0%, 10.4% and 2.6% respectively (Table 1). The neutral lipid content of D90G-19 was significantly greater than that of WT (P = 0.0093). On the contrary, the glycolipid and phospholipid contents of D90G-19 were lower than that of WT (P = 0.02 and P = 0.0052 respectively). In addition, the glycolipids contents were greater than phospholipid in the total lipid of WT and mutant D90G-19 under HL-N, which indicated the glycolipids were more prevalent in cell membrane of the mutant D90G-19 (P = 0.03) and might help the mutant to acclimate the phosphate-limiting growth conditions by providing more alternatives for phospholipids [35].

**Table 1:** Composition of total lipid in the *Desmodesmus* sp.

Lipid contents^a^	WT	D90G-19
neutral lipid (%)	78.14±1.52	86.97±1.28
Glycolipid (%)	14.85±0.97	10.39±0.88
phospholipid (%)	6.86±0.83	2.64±0.40
G:P^b^	2.18±0.15	3.96±0.26

a Microalgae cells were harvested after grown under HL-N (300–400 µmol photons m^−2^⋅s^−1^, nitrogen-depleted BG-11 medium with 4.25 mM NaNO_3_) for 8 days. Mean ± SE with three replicates.

b G:P: ratio of glycolipid to phospholipid.

**Figure 5:**
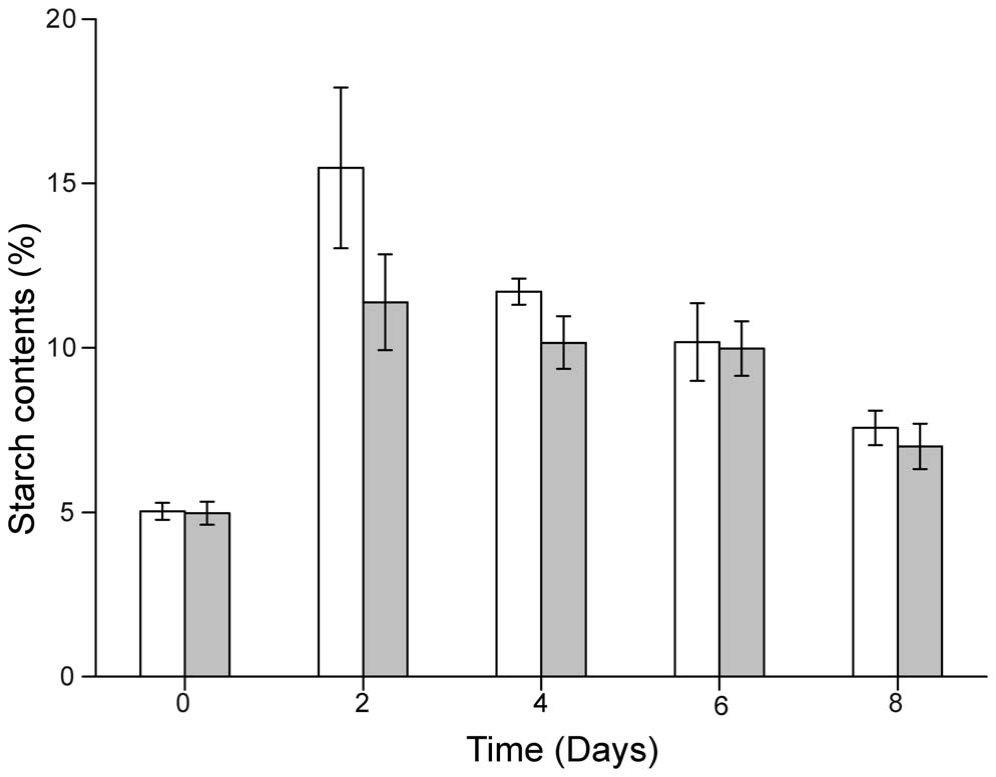
Starch contents of *Desmodesmus* sp. WT and D90G-19. The initial starch content was measured in algae cells grown under LL+N for 4 days. Afterwards, the cells were grown under HL-N (300–400 µmol photons m^−2^⋅s^−1^, nitrogen-limited BG-11 medium with 4.25 mM NaNO_3_). D90G-19: closed square; WT: open square.

Besides neutral lipid, starch is another major storage compound in many microalgae. A study showed that inhibition of starch synthesis resulted in a ten-fold increase of TAG in a starchless mutant of *C. reinhardtii* [8]. The total lipid content of the mutant D90G-19 was shown to be larger than that of the WT. In order to determine whether excess lipids accumulated in D90G-19 might be partially at the expense of starch, the starch contents of D90G-19 and WT were measured quantitatively. When grown under LL+N conditions for 4 days, WT and D90G-19 accumulated a basal amount of starch (about 5% of cells dry weight). After a shift to HL-N condition, WT and D90G-19 increased their starch contents by respectively 15.5% and 11.4% on day 2. Afterwards, the starch contents of WT and D90G-19 decreased to the same level (ca. 7% of dry weight) (Fig. 5) from the second day to the eighth day, but the lipid content of D90G-19 increased and was significantly higher than that of the WT at day 4, 6, and 8 (Fig. 4). Therefore, the additional neutral lipids in the D90G-19 should be synthesized via *de novo* fatty acid synthesis. Considering that the starch synthesis pathway in the D90G-19 was not blocked because the mutant D90G-19 can synthesize the same amounts of starch as the WT, it was possible that the conversion of starch to lipids may also occur in the cells [17].

### Characterization of Photosynthesis Efficiency of *Desmodesmus* sp

**Figure 6:**
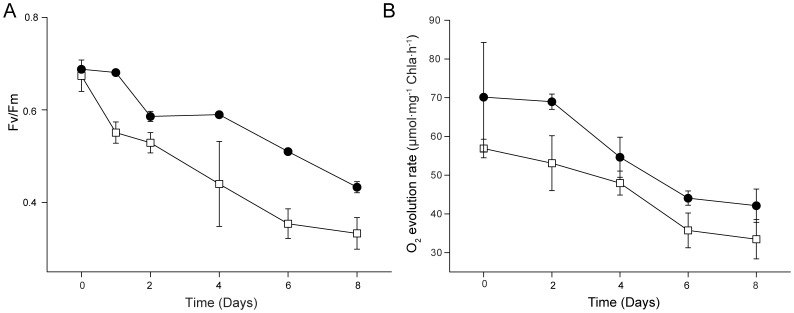
Photosynthesis efficiency of *Desmodesmus* sp. (A) Potential maximum quantum efficiency (Fv/Fm) and (B) oxygen evolution rate. WT and D90G-19 were grown under HL-N (300–400 µmol photons m^−2^⋅s^−1^, nitrogen-limited BG-11 medium with 4.25 mM NaNO_3_). D90G-19: closed circle; WT: open square.

Microalgae absorb light energy and convert it into chemical energy stored in the form of biochemical compounds such as carbohydrates, proteins and lipids. In order to elucidate the mechanism responsible for the higher lipid production by D90G-19 under stress conditions, the photosynthetic efficiency of the mutant was measured. The value of Fv/Fm reflected the potential maximum quantum efficiency of PSII. The Fv/Fm value was about 0.6–0.7 when WT and mutant D90G-19 were grown under optimal culture conditions (e.g. LL+N). Under stress (HL-N), the Fv/Fm value of WT decreased immediately whereas the decline of Fv/Fm in D90G-19 somewhat lagged behind, especially in the first day and from day 2 to day 4 (Fig. 6A). The results indicated that the mutant D90G-19 might tolerate higher light intensity and have the potential to use quanta more effectively at PSII reaction centers. Likewise, the O_2_ evolution rate of D90G-19 was faster than that of WT (Fig. 6B), which was consisted with the trend of Fv/Fm. The contents of chlorophyll a, chlorophyll b and carotenoids in WT and mutant D90G-19 were tested (Table 2). There was no significant difference in chlorophyll and carotenoid contents between the WT and D90G-19. The increase in potential quantum efficiency of PSII in D90G-19 may be the result from the changes in peripheral antenna complexes associated with PSII. Alternatively, the electron transport chain between PSI and PSII may be affected.

**Table 2:** Chlorophyll a, chlorophyll b and carotene contents in *Desmodesmus* sp.

Days	Chla (µg/mg)^a^		Chlb (µg/mg)		Carotenoids (µg/mg)		Chla/Chlb		(Chla+Chlb)/C^b^	
	WT	D90G-19	WT	D90G-19	WT	D90G-19	WT	D90G-19	WT	D90G-19
2	2.57±0.22	2.54±0.63	0.73±0.06	0.66±0.12	1.23±0.11	1.2±0.19	3.50	3.81	2.69	2.67
4	2.22±0.87	2.18±0.15	0.60±0.02	0.63±0.08	1.25±0.38	1.31±0.11	3.70	3.66	2.26	2.07
6	1.28±0.08	1.24±0.07	0.36±0.04	0.38±0.08	0.82±0.04	0.84±0.03	3.53	3.27	2.00	1.93
8	1.15±0.14	1±0.09	0.49±0.01	0.30±0.06	1.05±0.3	0.81±0.04	2.35	3.29	1.86	1.6

a Microalgae cells were grown under HL-N (300–400 µmol photons m^−2^⋅s^−1^, nitrogen-depleted BG-11 medium with 4.25 mM NaNO_3_). Mean ± SE with three replicates.

b Carotenoids.

It was reported that the partitioning of energy at PSII was disrupted by the decrease of starch synthesis in *Nicotina sylvestris* [36]. Blocking the competing starch synthesis pathway may facilitate carbon flux partitioning into lipid synthesis [8], but it may also lead to decrease of photosynthetic efficiency and growth impairment [37]. Our results suggested that the mutant exhibited higher photosynthetic efficiency under stress conditions, which resulted in more photosynthetically fixed carbon that could be redirected into neutral lipid biosynthesis.

### Fatty Acid Profiles of the Different Classes of Glycerolipids in the *Desmodesmus* sp. WT and Mutant under HL-N

The fatty acid acyl profiles of total lipid, neutral lipid, glycolipid and phospholipid in the mutant D90G-19 were similar to those of the WT (Table 3). As for the unsaturation of fatty acid acyl in lipids, there was no significant difference between the WT and mutant D90G-19, although the lipid unsaturation of D90G-19 was slightly higher. Among the three major classes of glycerolipids, phospholipids contained the highest amounts of unsaturated acyl moieties contributing likely to the fluidity of cell membranes. However, the majority of membrane glycerolipids are glycolipid rather than phospholipid, MGDG and DGDG are the predominant species of chloroplast membrane under HL-N. There are two pathways for the assembly of lipid precursors in plant and microalgae: a *de novo* lipid assembly pathway in the plastid and a pathway located at the endoplasmic reticulum (ER) [38]. Glycerolipids synthesized by the ER pathway have a different molecular species composition (18-carbon fatty acids in the *sn*-2 position of the glycerol backbone) from those produced by the plastid pathway (16-carbon fatty acids in *sn*-2) [39]. The results (table 3) showed that 18-carbon fatty acids acyl chains were predominant in three classes of glycerolipids, especially in neutral lipids, implying that the ER was the major site for the synthesis of storage neutral lipid in *Desmodesmus*, which was similar to many other plants and algae [7], [34], [38], [40].

**Table 3:** Fatty acid compositions of individual lipid classes in *Desmodesmus* sp.^d^.

FA	Total Lipid		Neutral lipid		Glycolipid		Phospholipid	
	WT	D90G-19	WT	D90G-19	WT	D90G-19	WT	D90G-19
C16∶0	17.08±0.65	15.03±0.43	17.03±0.44	15.15±0.60	16.65±1.20	13.55±1.05	16.94±2.92	15.42±1.48
C16∶1	3.35±0.67	3.62±0.54	3.45±1.21	4.02±0.82	4.74±2.23	5.50±1.09	3.77±0.13	3.54±0.34
C16∶2	2.47±0.37	3.08±0.27	2.29±0.70	3.05±0.40	3.34±1.01	4.20±0.33	7.02±2.99	9.42±1.21
C16∶3	3.09±0.69	3.82±0.44	2.82±0.06	3.21±0.46	3.91±0.46	4.13±0.64	7.03±1.30	7.58±1.21
C16∶4	1.47±0.65	1.53±0.28	1.11±0.21	1.14±0.22	2.26±0.88	1.96±0.42	6.45±3.53	6.03±1.20
C18∶0	9.73±2.68	9.60±1.23	10.62±1.93	10.74±0.20	9.15±0.71	7.08±0.57	5.79±1.24	4.27±1.35
C18∶1	44.81±1.42	45.05±1.21	46.23±2.04	46.86±0.66	39.32±2.67	40.88±1.29	17.42±2.52	17.06±0.92
C18∶2	7.99±1.04	7.91±1.14	6.64±0.90	6.48±0.73	9.16±0.67	10.30±1.43	13.88±1.01	13.37±0.87
C18∶3	9.38±1.78	9.58±0.98	7.74±0.21	8.28±0.67	11.11±1.39	11.93±1.50	22.95±4.14	24.67±3.95
C20∶0	0.85±0.24	0.59±0.08	0.88±0.13	0.65±0.03	ND^c^	ND	ND	ND
C20∶1	0.90±0.13	0.70±0.09	0.84±0.38	0.60±0.01	ND	ND	ND	ND
C16:C18	0.38	0.38	0.37	0.37	0.45	0.42	0.69	0.71
unsatd^a^	73.45	75.29	71.14	73.63	73.84	78.89	78.52	81.68
▽/mol^b^	1.13	1.18	1.05	1.10	1.23	1.31	1.79	1.87

a percentage of unsaturated fatty acids in total fatty acids.

b the degree of fatty acid unsaturation [41].

c not detected.

d Microalgae were harvested after grown under HL-N (300–400 µmol photons m^−2^⋅s^−1^, nitrogen-depleted BG-11 medium with 4.25 mM NaNO_3_) for 8 days. Mean ± SE with three replicates.

### Conclusions

This work represented the first attempt to use heavy carbon ions (^12^C^6+^) to induce mutagenesis of oleaginous microalgae to obtain mutants with enhanced lipid production potential. The mutation rate was about 20–30% when *Desmodesmus* cells were treated with 60–120 Gy heavy-ion beam, and a wide spectrum of phenotypes in lipid contents and photosynthetic efficiency was observed. Of numerous genuine mutants obtained, D90G-19 exhibited 20.6% higher lipid productivity than WT when they were cultivated in the column bioreactors, likely owing to an improved quantum efficiency of photosynthesis under stress conditions.

## Supporting Information

Figure S1 **Biomass concentration of** ***Desmodesmus*** **sp. WT (open squares) and D90G-19 (closed square) when cultivated in nitrogen-limited medium with 4.25 mM NaNO_3_ and high light illumination (300–400 µmol photons m^−2^⋅s^−1^) in a column photobioreactor.**
(DOC)

Figure S2 **Biomass (A) and total lipid content (B) of** ***Desmodesmus*** **sp. WT (open square) and D90G-19 (closed square) when cultivated in nitrogen-limited medium with 4.25 mM NaNO_3_ and high light illumination (300–400 µmol photons m^−2^⋅s^−1^) in a 15 L panel photobioreactor.** The total lipid contents of mutant D90G-19 was significantly higher than WT at day 10 (P = 0.026) and day 12 (P = 0.036).
(DOC)

Table S1 **Fv/Fm value and lipid contents of WT and 20 mutants.** Mean ± SE with three replicates.
(DOC)
