# Supplementary material for: Enhanced Lipid Productivity and Photosynthesis Efficiency in a Desmodesmus sp. Mutant Induced by Heavy Carbon Ions
Source: PLoS One. 2013 Apr 9;8(4):e60700. doi: 10.1371/journal.pone.0060700 (PMC3621885; doi:10.1371/journal.pone.0060700)
Supplement: Figure S1 — Biomass concentration of Desmodesmus sp. WT (open squares) and D90G-19 (closed square) when cultivated in nitrogen-limited medium with 4.25 mM NaNO3 and high light illumination (300–400 µmol photons m−2⋅s−1) in a column photobioreactor. (DOC) [file pone.0060700.s001.doc]

**Supplementary data caption**

Supplementary figure S1. Biomass concentration of *Desmodesmus* sp. WT (open squares) and D90G-19 (closed square) when cultivated in nitrogen-limited medium with 4.25 mM NaNO_3_ and high light illumination (300-400 μmol photons m^-2^∙s^-1^) in a column photobioreactor.

Figure S1
