# Supplementary material for: Enhanced Lipid Productivity and Photosynthesis Efficiency in a Desmodesmus sp. Mutant Induced by Heavy Carbon Ions
Source: PLoS One. 2013 Apr 9;8(4):e60700. doi: 10.1371/journal.pone.0060700 (PMC3621885; doi:10.1371/journal.pone.0060700)
Supplement: Figure S2 — Biomass (A) and total lipid content (B) of Desmodesmus sp. WT (open square) and D90G-19 (closed square) when cultivated in nitrogen-limited medium with 4.25 mM NaNO3 and high light illumination (300–400 µmol photons m−2⋅s−1) in a 15 L panel photobioreactor. The total lipid contents of mutant D90G-19 was significantly higher than WT at day 10 (P = 0.026) and day 12 (P = 0.036). (DOC) [file pone.0060700.s002.doc]

**Supplementary data caption**

Supplementary figure S2. Biomass (A) and total lipid content (B) of *Desmodesmus* sp. WT (open square) and D90G-19 (closed square) when cultivated in nitrogen-limited medium with 4.25 mM NaNO_3_ and high light illumination (300-400 μmol photons m^-2^∙s^-1^) in a 15 L panel photobioreactor. The total lipid contents of mutant D90G-19 was significantly higher than WT at day 10 (P=0.026) and day 12 (P=0.036).

Figure S2A

Figure S2B
