# Supplementary material for: Enhanced Lipid Productivity and Photosynthesis Efficiency in a Desmodesmus sp. Mutant Induced by Heavy Carbon Ions
Source: PLoS One. 2013 Apr 9;8(4):e60700. doi: 10.1371/journal.pone.0060700 (PMC3621885; doi:10.1371/journal.pone.0060700)
Supplement: Table S1 — Fv/Fm value and lipid contents of WT and 20 mutants. Mean ± SE with three replicates. (DOC) [file pone.0060700.s003.doc]

**Supplementary data caption**

Supplementary Table 1. Fv/Fm value and lipid contents of WT and 20 mutants. Mean ± SE with three replicates.

| strain^a^ | Fv/Fm | Lipid Contents (%) |
| --- | --- | --- |
| WT | 0.453±0.002 | 36.2±0.764 |
| D90G-h1 | 0.610±0.02 | 46.3±2.346 |
| D60G-392 | 0.629±0.02 | 45.6±2.793 |
| D60G-251 | 0.510±0.052 | 41.6±1.498 |
| D60G-229 | 0.473±0.033 | 45.5±0.721 |
| D90G-181 | 0.425±0.030 | 36.2±2.386 |
| D30G-7 | 0.526±0.016 | 40.1±2,212 |
| D60G-370 | 0.420±0.054 | 38.4±2.775 |
| D30G-273 | 0.520±0.013 | 40.3±1.069 |
| D30G-84 | 0.516±0.015 | 40.5±2.052 |
| D90G-188 | 0.592±0.025 | 47.7±1.680 |
| D60G-45 | 0.598±0.028 | 47.0±2.470 |
| D60G-216 | 0.517±0.045 | 40.1±2.386 |
| D30GG-106 | 0.412±0.015 | 40.0±6.717 |
| D90G-329 | 0.128±0.013 | 16.5±2.011 |
| D90G-65 | 0.241±0.034 | 23.1±1.922 |
| D30G-44 | 0.405±0.030 | 30.9±3.656 |
| D90G-315 | 0.423±0.029 | 31.7±2.227 |
| D30G-206 | 0.252±0.018 | 25.5±2.401 |
| D30G-69 | 0.302±0.013 | 30.1±2.914 |
| D90G-19 | 0.59±0.006 | 45.69±1.76 |

a. The WT and nineteen mutants identified as both PEM and LOM by Imaging-PAM and Nile red staining assay were cultivated under stress conditions HL-N (300-400 μmol photons m^-2^∙s^-1^, nitrogen-limited BG-11 medium with 4.25 mM NaNO_3_). Fv/Fm value was measured on day 4, and lipid content was analyzed on day 8.
